# Modelling quarantine effects on SARS-CoV-2 epidemiological dynamics in Chilean communes and their relationship with the Social Priority Index

**DOI:** 10.7717/peerj.14892

**Published:** 2023-03-10

**Authors:** Dino G. Salinas, M. Leonor Bustamante, Mauricio O. Gallardo

**Affiliations:** 1 Centro de Investigación Biomédica, Facultad de Medicina, Universidad Diego Portales, Santiago, Chile; 2 Human Genetics Program, Biomedical Sciences Institute, Faculty of Medicine, Universidad de Chile, Santiago, Chile; 3 Department of Psychiatry and Mental Health, North Division, Faculty of Medicine, Universidad de Chile, Santiago, Chile

**Keywords:** Reproduction number, COVID-19, Differential equations, Social inequality, Quarantine, Lockdown, SARS-CoV-2, Social distancing, Social priority index, Confirmed infected cases

## Abstract

**Background:**

An epidemiological model (susceptible, un-quarantined infected, quarantined infected, confirmed infected (SUQC)) was previously developed and applied to incorporate quarantine measures and calculate COVID-19 contagion dynamics and pandemic control in some Chinese regions. Here, we generalized this model to incorporate the disease recovery rate and applied our model to records of the total number of confirmed cases of people infected with the SARS-CoV-2 virus in some Chilean communes.

**Methods:**

In each commune, two consecutive stages were considered: a stage without quarantine and an immediately subsequent quarantine stage imposed by the Ministry of Health. To adjust the model, typical epidemiological parameters were determined, such as the confirmation rate and the quarantine rate. The latter allowed us to calculate the reproduction number.

**Results:**

The mathematical model adequately reproduced the data, indicating a higher quarantine rate when quarantine was imposed by the health authority, with a corresponding decrease in the reproduction number of the virus down to values that prevent or decrease its exponential spread. In general, during this second stage, the communes with the lowest social priority indices had the highest quarantine rates, and therefore, the lowest effective viral reproduction numbers. This study provides useful evidence to address the health inequity of pandemics. The mathematical model applied here can be used in other regions or easily modified for other cases of infectious disease control by quarantine.

## Introduction

Mathematical models of the spread of infectious diseases can be very useful for monitoring and controlling epidemics when such models use and predict the number of infected people. These figures are frequently reported by health authorities during a pandemic, as in the current coronavirus (COVID-19) pandemic, which is caused by the severe acute respiratory syndrome coronavirus 2 (SARS-CoV-2) (Synowiec et al., 2021). In many countries, periodic reports on the total number of confirmed cases by region have been issued, and these reports have helped the population follow the pandemic dynamics. However, unlike the model that we propose and apply here, not all mathematical models of academic interest meet the practical requirement of fitting the data included in official epidemiological reports.

Dynamic models, such as Susceptible-Infectious-Removed (SIR), Susceptible-Exposed-Infectious-Removed (SEIR), and others (Fisman, Khoo & Tuite, 2014; Stehlé et al., 2011; Yang & Wang, 2020), are difficult to apply to SARS-CoV-2 because of three main characteristics: the relatively long incubation period, artificial factors (medical resources and quarantines), and variations in the efficiency of confirmation methods. A generalized SIR model with constant time delays has been applied for COVID-19 data of three regions of Chile, determining a reproducible parameter estimation, and such that the methodology could be applied to any country (Cumsille et al., 2022). For COVID-19, an extended SEIR model has also been used, including asymptomatic infected individuals and interspecies infection (Chen et al., 2020). Alternatively, in a more simplified epidemiological system, but addressing the quarantines with a low number of fitting parameters, an appropriate model to study the SARS-CoV-2 propagation dynamics is the SUQC model (Zhao & Chen, 2020), which was applied to data of the total number of daily confirmed cases recorded in official Chinese reports from January and February 2020. Using this model, it was determined the values of three epidemiological parameters useful for epidemic control, monitoring, intervention, and evaluation: the reproduction number (Ridenhour, Kowalik & Shay, 2013; Wallinga & Lipsitch, 2007), quarantine rate, and confirmation rate. Below, we review this model and then propose its generalization and subsequent application to some Chilean communes. In addition to providing an analytical relationship between the “effective reproduction number” and the “quarantine rate”, another important advantage of our extended model is that, compared to others, only a few adjustment parameters are necessary. These simple features facilitate the study of causal relationships with external factors, such as health policies or social conditions, as well as the comparative study between different localities.

For the SUQC model (Zhao & Chen, 2020), and assuming a commune of *N* inhabitants, the following types of individuals were considered: *S*, susceptible to infection; *U*, infected, unquarantined, infectious (unlike *E* in the SEIR model) and either presymptomatic or symptomatic; *Q*, infected and quarantined, and therefore, non-infectious, deriving from a *U*, hospitalized or isolated; and *C*, confirmed infected case. The removed individuals, *R*, of the SIR and SEIR models is disregarded in SUQC model, which assumes there is no recovery or death. The total number of individuals of type *S*, *U*, *Q*,  and *C* at time *t* is indicated as *S*(*t*), *U*(*t*), *Q*(*t*), and *C*(*t*), respectively.

The epidemiological parameters of the SUQC model are as follows:

*α*,  the number of individuals infected by an unquarantined individual per day, the infection rate ($S\rightarrow ^{\alpha }U$); *αϵ*[0, ∞).

*γ*, the quarantine rate ($U\rightarrow ^{\gamma }Q$); *γϵ*[0, 1].

and *β*, the confirmation rate of *Q* as *C* by mean of conventional method (*e.g.*, laboratory diagnosis) or by subsequent additional tests, *i.e.,* the total confirmation rate ($Q\rightarrow ^{\beta }C$); *βϵ*[0, 1].

In the complete SUQC model, *U* goes directly to C (*U* → *C*), or throug *Q* (*U* → *Q* → *C*). However, *U* → *C* may not be noted since it is indistinguishable from *U* → *Q* → *C* with zero delay time during *Q* → *C*. Therefore, the simplified SUQC model is defined by the following system of ordinary differential equations (Zhao & Chen, 2020):

(1) \begin{eqnarray*} \frac{dS(t)}{dt} & =-\alpha U(t)S(t)/N\end{eqnarray*}

(2) \begin{eqnarray*} \frac{dU(t)}{dt} & =\alpha U(t)S(t)/N-\gamma U(t)\end{eqnarray*}

(3) \begin{eqnarray*} \frac{dQ(t)}{dt} & =\gamma U(t)-\beta Q(t)\end{eqnarray*}

(4) \begin{eqnarray*} \frac{dC(t)}{dt} & =\beta Q(t)\end{eqnarray*}

Note that, according to Eq. (4), *C*(*t*) corresponds to the total number of cases of infection confirmed until time *t* (*i.e.,* the cumulative number of cases).

Proposing a generalization of the previous model, we have modeled the spread of SARS-CoV-2 in some Chilean communes of the Metropolitan Region (including Santiago, the capital of the country), over the period of a few months during 2020, under different quarantine measures, depending on whether they were spontaneous or imposed by the health authority. The generalized model proposed by us, SUQCR, unlike the simplified SUQC model (hereafter only SUQC model), includes recovered individuals (*R*) depending on the recovery rate of infectious individuals (*U* and *Q*). Dead individuals are not included in the model. In addition to proposing the SUQCR model, this study aimed to demonstrate its applicability, calculating and comparing epidemiologically relevant fitting parameters of each commune according to its quarantine measures. It also aimed to assess whether these results have any correlation with the Social Priority Index (SPI), a parameter that indicates the social priority in each commune studied (Región Metropolitana De Santiago. Índice De Prioridad Social De Comunas 2020. Seremi de Desarrollo Social y Familia Metropolitana). It has been observed worldwide that the most vulnerable populations have endured a greater impact of COVID-19 worldwide (Wang & Tang, 2020), however it is unclear whether this is due to inequities in access to healthcare, prevalence of factors predisposing to severe illness or other variables (Gu et al., 2020; Mena et al., 2021). Thus, in the present study we sought to analyze specifically the relationship between socioeconomic factors and the effectiveness of a nonpharmacological intervention on the prevention of SARS-CoV-2 spread. We believe that this study contributes new scientific evidence for addressing health inequity during a pandemic.

## Methodology

### Definition of the SUQCR model

**Figure 1:**
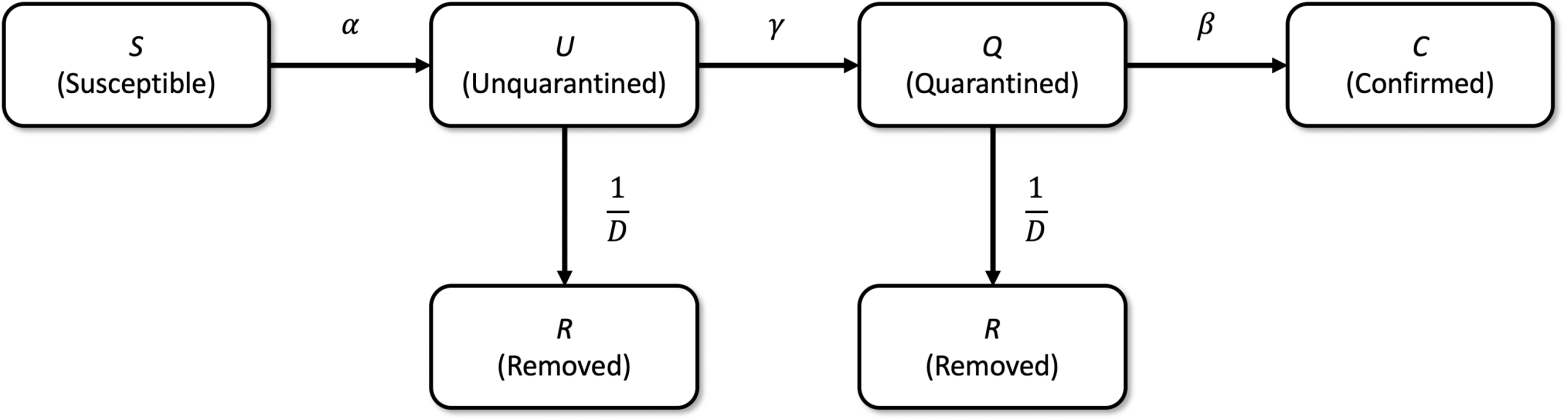
Schematic of the SUQCR model. The proposed model in this study (Eqs. (5), (6), (7) and (8)) is a generalization of the SUQC model (Zhao & Chen, 2020), which includes *R* individuals, who are no longer infectious, are considered recovered, and derive from *Q* or *U*, at a rate of 1/*D*.

We define the SUQCR model by including removed individuals (*R*) in the simplified SUQC model. However, *R* representing individuals who are no longer infectious, who are considered recovered, and who derive from *Q* or *U*, at a rate of 1/ *D*; as such, the mean time of recovery from the disease is *D*, in days. The factor 1/ *D* does not apply to individuals *C* because they correspond to cases counted as confirmed at any time up to *t*. Thus, the SUQCR model that we propose here as a generalization of the SUQC model is defined in the scheme of Fig. 1 and by the following system of ordinary differential equations:

(5) \begin{eqnarray*} \frac{dS(t)}{dt} & =-\alpha U(t)S(t)/N\end{eqnarray*}

(6) \begin{eqnarray*} \frac{dU(t)}{dt} & =\alpha U(t)S(t)/N- \left( \gamma +1/D \right) U(t)\end{eqnarray*}

(7) \begin{eqnarray*} \frac{dQ(t)}{dt} & =\gamma U(t)- \left( \beta +1/D \right) Q(t)\end{eqnarray*}

(8) \begin{eqnarray*} \frac{dC(t)}{dt} & =\beta Q(t)\end{eqnarray*}

Note that $N=S \left( t \right) +U \left( t \right) +Q \left( t \right) +C \left( t \right) +R(t)$. We assume that *R*(*t* = 0) ≈ 0.

To demonstrate the relationship between the SUQC and SUQCR models, note that Eqs. (5) and (8) are the same as Eqs. (1) and (4), respectively. In addition, Eqs. (6) and (7) converge to Eqs. (2) and (3). respectively, when *D* ≫ 1. This demonstrates that SUQCR is a generalization of SUQC.

The model studied did not consider the latency period of COVID-19. Through meta-analysis studies, it has been determined that the latency period of SARS-CoV-2 is about 5 days on average, within a wider range (Grudlewska-Buda et al., 2021). Considering that here we have used *C*(*t*) values reported on average at 3 days, with 25% standard deviation, in a range of 2 to 5 days, we believe that data updated daily would be more appropriate to study the effect of the latency period.

### Study communes

The modeled data were the values of *C*(*t*) recorded in each of the 32 communes of the province of Santiago, in the Metropolitan Region of Chile. The data from each of these communes were grouped into two consecutive stages. The first stage (stage 1) is without quarantine imposed by the health authorities. Given the influence of national and international news about the pandemic, the constant advice of subject-matter experts and quarantines previously imposed in some communes of the country, a fraction of the population most likely followed some degree of quarantine and other social distancing measures in stage 1, in addition to hygiene protocols and the use of a masks (Howard et al., 2021), albeit voluntarily. In the second stage (stage 2), quarantine was officially imposed by the health authorities, mandating compliance with strict social distancing protocols and other self-care practices.

### Total number of confirmed cases of infection (***C***(***t***)) by commune during the study period

The values of *C*(*t*) for each of the 32 communes are from the epidemiological records of the period (corresponding to stages 1 and 2 for all communes) spanned from March 30 to June 15, 2020 (Ministerio de Ciencia, Technologia, Conocimiento e Innovacion, 2020).

### Calculation of epidemiological parameters of the SUQCR model

The value of *α* could no longer be measured in pure form through an exponential model simpler than the model that we applied here because most of the population already had enough information to adopt some self-care measures by the time the pandemic arrived in Chile. However, such a simple exponential approximation was used in China with the first data collected in Wuhan, where the first-ever cases were recorded. As a result, the value of the infection rate, *α* = 0.2967, calculated for Wuhan from January 20 to 27, 2020, was used in the SUQC model, as applied to a few other regions of China. In this model, the reproduction number, ℜ, was calculated considering that fluctuations in *α* could be compensated by fluctuations in *γ* because ℜ = *α*/*γ* (Zhao & Chen, 2020). However, when *γ* = 0 this ℜ value is indeterminate.

For the SUQCR model, defining $\mathfrak{R}\equiv \alpha / \left( \gamma +1/D \right) $, assuming that susceptible individuals almost do not decrease compared to the total population (*S*(*t*) ≈ *N*), and from Eq. (6), we have

(9) \begin{eqnarray*}U(t)=U(t=0){e}^{ \left( \gamma +1/D \right) \left( \mathfrak{R}-1 \right) t}\end{eqnarray*}

In this case, ℜ corresponds to an effective reproduction number, due to the quarantine effects represented by *γ*, such that ℜ < ℜ_*basic*_, with ℜ_*basic*_ = *αD* (the basic number of reproduction) (Ridenhour, Kowalik & Shay, 2013), using *D* = 14 days (Byrne et al., 2020) and the aforementioned value of *α* (Zhao & Chen, 2020).

Each quarantine stage, 1 and 2 (*i.e.,* voluntary and imposed), has the following time series: a time series of *C*(*t*), denoted by **C**, derived from the study period, and a theoretical time series, denoted by $\hat {\mathbf{C}}$, corresponding to the same days, determined by numerical integration of the Eqs. (5), (6), (7) and (8) system using the Runge–Kutta method and described as a function $f \left( \right) $ of the fitting parameters.

(10) \begin{eqnarray*}\hat {\mathbf{C}}=f \left( \beta ,\gamma ,U(t=0),Q(t=0) \right) \end{eqnarray*}

The fitting parameters *β*, *γ*, *U*(*t* = 0) and *Q*(*t* = 0) were calculated by minimizing the error function *err*, which is equal to the mean squared deviation between the empirical and theoretical time series data:

(11) \begin{eqnarray*}err \left( \beta ,\gamma ,U(t=0),Q(t=0) \right) ={ \left\| \mathbf{C}-\hat {\mathbf{C}} \right\| }^{2}\end{eqnarray*}

In fitting the parameters, to minimize *err* (Eq. 11), we alternatively used the MATLAB function *fminsearch* or *fmincon* (Birgin & Gentil, 2012) to optimize the fit of the model: fminsearch finds a minimum of unconstrained multivariable function. Instead, *fmincon* finds a minimum of constrained nonlinear multivariable function. In general, both functions were useful when used in the data adjustments to the model, thus obtaining two sets of adjustment parameters per stage and per commune. However, for each commune, we select the best adjustment results obtained.

The data and programs used are available as supplementary material.

### Study of the estimated parameters by commune

After applying the SUQCR model to each commune and in each quarantine stage, the goodness-of-fit parameters were recorded, and the values of *β*, *γ* and ℜ were analyzed for stages 1 and 2. To compare the mean values per quarantine stage of epidemiologically relevant parameters (*i.e.,* quarantine rate, *γ*; confirmation rate, *β*; and reproduction number, ℜ) of the 32 communes of the province of Santiago, Student’s *t*-test was applied to the mean values of two paired samples, in two-tailed tests. Specifically, in the two paired samples the values of each parameter in one commune in stage 1 is compared with the new value in stage 2 for the same parameter and commune. Moreover, the correlations between *γ* and the social priority index SPI were studied. The SPI is used by the Metropolitan Regional Government to support the communes with the greatest relative deficiencies, and it is an indicator developed for the Chilean Secretary of Social Development to assist in the design and assessment of public policies (Seremi de Desarrollo Social y Familia Metropolitana, 2020). The method for its estimation has changed from when it was initially launched in 1995, as the main determinants of the quality of life of the inhabitants of the Metropolitan region change along the years. However, its focus remains the three most relevant dimensions of social development (income, education, and health), and its numerical value is meant to establish a comparison between the relative standard of living of the people from each commune and targeting the resources for solving social needs. Each dimension has 2–4 indicators that are scored from 0–100, where 0 is the lowest priority and 100 is the worst relative situation or highest priority. The final score is calculated as the weighted mean of each indicator.

## Results

**Figure 2:**
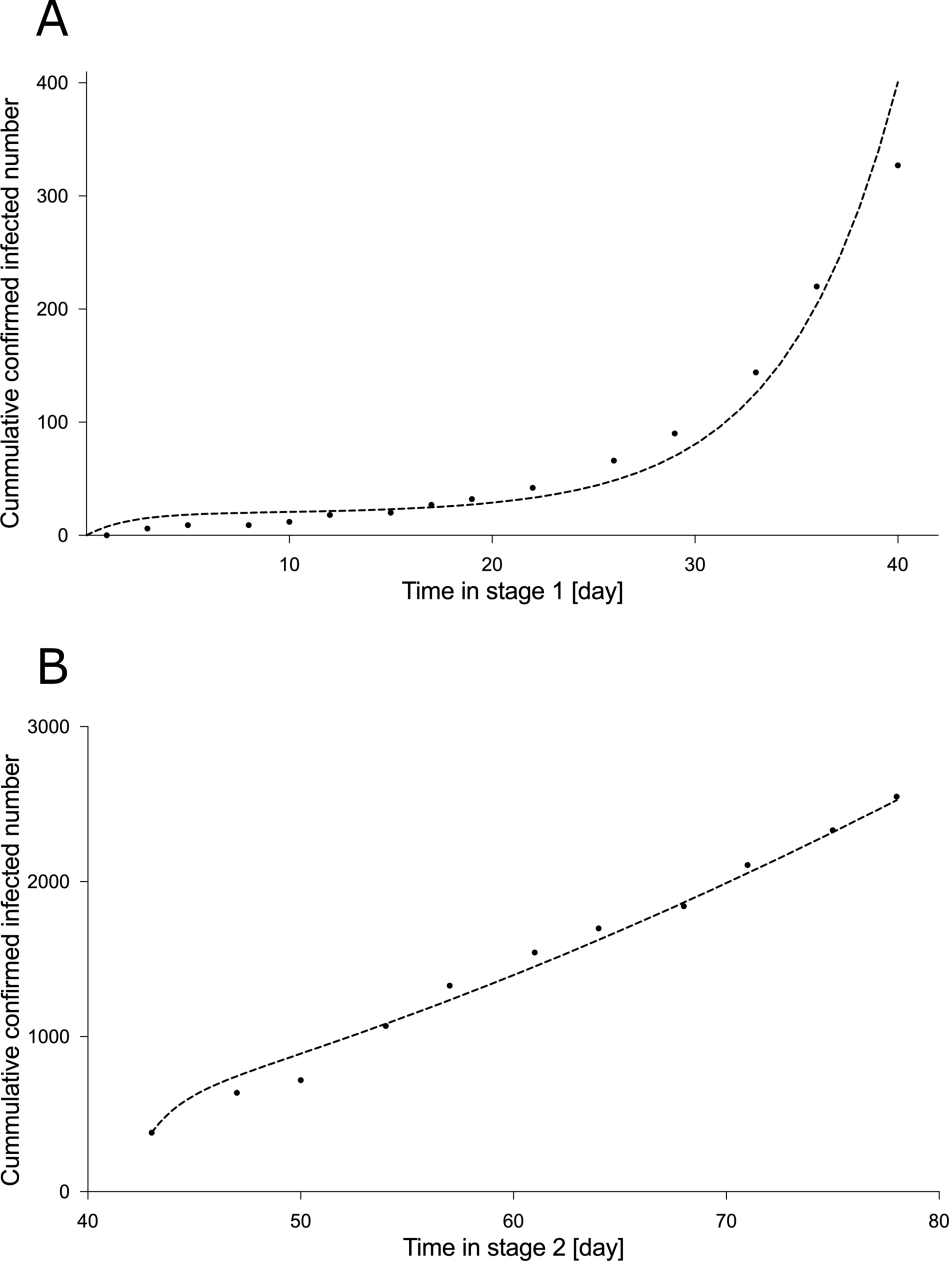
Curve fitting of the SUQCR model of the San Ramón commune. The used data are the total confirmed cases of the San Ramón commune, in the Metropolitan Region of Chile, spanning from March 30 to June 15, 2020, and according to the timeframe in Supplementary file S2. The goodness-of-fit results are outlined in Table 1. (A) Without quarantine imposed by the health authorities (stage 1). (B) With quarantine imposed by the health authorities (stage 2). The vertical legend corresponds to the total number of confirmed cases of infection (*C*(*t*)).

We applied the SUQCR mathematical model to data of total confirmed cases of infection (*C*(*t*)) during approximately three months, in stages 1 and 2, for each of the 32 study communes (Ministerio de Ciencia, Technologia, Conocimiento e Innovacion, 2020). More specifically, data were spanned from March 30 to June 15, 2020, and according to the timeframe in Supplementary File S2. Thereby calculating the Pearson’s correlation coefficient, *r*, and the corresponding fitting parameters for each commune. As a case in point, Table 1 outlines the parameters of the SUQCR model for 3 communes, and Fig. 2 shows a typical curve fitting of the model to the total number of confirmed cases reported in a commune.

**Table 1:** Goodness-of-fit results of some epidemiologically relevant parameters of three Chilean communes studied. As defined by Eqs. (5), (6), (7) and (8), the model was fitted to the records of the number of total confirmed infected cases (*C*(*t*)), which were sourced from the Ministry of Health. The start and end dates of each stage are indicated. Data were spanned from March 30 to June 15, 2020, and according to the timeframe in Supplementary File S2.

		San Ramón (Population: 86,510)		Recoleta (Population: 190,075)		Santiago Centro (Population: 503,147)	
		Stage I (30-March-2020 to 8-May-2020)	Stage II (11-May-2020 to 15-June-2020	Stage I (30-March-2020 to 5-May-2020)	Stage II (8-May-2020 to 15-June-2020)	Stage I (30-March-2020 to 4-May-2020)	Stage II (8-May-2020 to 15-June-2020)
***U***(***t*** = **0**)	Calculated parameter	1.7708	227.6755	12.8968	470.0697	90.9906	1,795.5073
***Q***(***t*** = **0**)	Calculated parameter	22.9903	295.4115	0.6689	110.4116	0.0000	154.6483
*β* [day^−1^]	Calculated parameter	0.4208	0.6370	0.6410	0.6102	0.3047	0.1096
*γ* [day^−1^]	Calculated parameter	0.0391	0.2019	0.1060	0.2042	0.1740	0.2220
ℜ	Eq. 9	2.6832	1.0856	1.6723	1.0763	1.2091	1.0113
*r* ^2^	Square of the Pearson’s correlation coefficient	0.9820	0.9912	0.9836	0.9909	0.9762	0.9990

Table 2 outlines epidemiologically relevant parameters for the 32 communes in stages 1 and 2. There were 63 goodness-of-fit with *r*^2^ >0.90, and one with *r*^2^ ≈ 0.85. In all communes, the ℜ decreased from stage 1 to stage 2.

**Table 2:** Goodness-of-fit of the parameters assessed for the SUQCR model (Eqs. 5-8). In total, 32 communes were studied in the province of Santiago, Chile. The continuous interval of days corresponding to stages 1 and 2 was extended for all communes from March 30 to June 15, 2020 (Supplementary File S2). The number of inhabitants (*N*) and the social priority index (SPI) are also shown for each commune. Values of SPI are for communes in 2020, available as IPS 2020 in the report of Regional Ministerial Secretariat for Social Development and Metropolitan Family (Seremi de Desarrollo Social y Familia Metropolitana, 2020).

			**Stage 1**				**Stage 2**			
**City**	***N***	**SPI**	*γ* [day^−1^]	*β*[day^−1^]	ℜ	*r* ^2^	*γ*[day^−1^]	*β*[day^−1^]	ℜ	*r* ^2^
**Lo Prado**	104,403	77.71	0.1512	0.8971	1.3328	0.9937	0.1611	0.3124	1.2758	0.9942
**Quinta Normal**	136,368	73.17	0.1385	0.4473	1.4133	0.9953	0.1672	0.8299	1.2435	0.9983
**La Granja**	122,557	76.37	0.1281	0.7785	1.4873	0.9914	0.1684	0.3836	1.2372	0.9526
**Renca**	160,847	72.85	0.1321	0.4428	1.4581	0.9967	0.1693	0.7057	1.2328	0.9952
**Estación Central**	206,792	71.67	0.1569	0.9234	1.2995	0.9959	0.1696	0.3635	1.2310	0.9777
**Cerro Navia**	142,465	85.91	0.1598	0.3463	1.2830	0.9976	0.1730	0.4011	1.2139	0.9958
**El Bosque**	172,000	80.97	0.1301	0.2469	1.4723	0.9852	0.1742	0.9965	1.2078	0.9984
**Independencia**	142,065	72.2	0.1006	0.5141	1.7244	0.9983	0.1824	0.9072	1.1689	0.9901
**La Pintana**	189,335	89.29	0.1415	0.5240	1.3935	0.9980	0.1829	0.2711	1.1668	0.9991
**Pudahuel**	253,139	67.64	0.1401	0.2495	1.4027	0.9986	0.1840	0.2644	1.1614	0.9942
**Pedro Aguirre Cerda**	107,803	75.65	0.1152	0.9151	1.5901	0.9567	0.1871	0.0545	1.1478	0.9967
**Cerrillos**	88,956	67.81	0.0476	0.4451	2.4917	0.9598	0.1877	0.4365	1.1448	0.9951
**La Cisterna**	100,434	70.21	0.1398	0.9053	1.4049	0.9919	0.1922	0.4830	1.1254	0.9970
**Lo Espejo**	103,865	88.83	0.1660	1.0000	1.2494	0.9902	0.1954	0.5875	1.1120	0.9974
**Peñalolén**	266,798	66.19	0.0488	0.8321	2.4680	0.9255	0.1993	0.4479	1.0958	0.9980
**Ñuñoa**	250,192	40.96	0.0950	0.7007	1.7830	0.9162	0.1997	0.5543	1.0942	0.9993
**Maipú**	578,605	60.86	0.1711	0.6727	1.2232	0.9822	0.2013	0.5732	1.0879	0.9989
**San Miguel**	133,059	56.63	0.1524	0.4472	1.3255	0.9911	0.2018	0.3575	1.0859	0.9987
**San Ramón**	86,510	83.5	0.0391	0.4208	2.6832	0.9820	0.2019	0.6370	1.0856	0.9912
**Conchalí**	139,195	79.87	0.1197	0.4339	1.5525	0.9931	0.2029	0.9958	1.0817	0.9936
**Recoleta**	190,075	76.6	0.1060	0.6410	1.6723	0.9836	0.2042	0.6102	1.0763	0.9909
**Macul**	134,635	57.63	0.1217	0.3818	1.5366	0.9955	0.2053	0.5144	1.0721	0.9995
**La Reina**	100,252	38.35	0.1575	0.2767	1.2963	0.9944	0.2083	0.4125	1.0608	0.9985
**La Florida**	402,433	64.22	0.1637	0.1078	1.2616	0.9823	0.2088	0.2390	1.0588	0.9992
**San Joaquín**	103,485	76.87	0.1373	0.1117	1.4212	0.9891	0.2119	0.4355	1.0471	0.9958
**Las Condes**	330,759	11.64	0.1805	0.5566	1.1775	0.9376	0.2160	0.4729	1.0324	0.9987
**Quilicura**	254,694	58.69	0.0323	0.8372	2.8605	0.8521	0.2177	0.3183	1.0262	0.9976
**Santiago Centro**	503,147	55.2	0.1740	0.3047	1.2091	0.9762	0.2220	0.1096	1.0113	0.9990
**Lo Barnechea**	124,076	35.08	0.1512	0.4810	1.3327	0.9937	0.2256	0.4664	0.9989	0.9998
**Huechuraba**	112,528	56.6	0.1439	0.9918	1.3782	0.9896	0.2279	0.4051	0.9911	0.9970
**Providencia**	157,749	24.91	0.1607	0.4301	1.2781	0.9864	0.2316	0.3368	0.9790	0.9997
**Vitacura**	96,774	7.94	0.1249	0.5546	1.5115	0.9812	0.2386	0.4164	0.9571	0.9980

**Figure 3:**
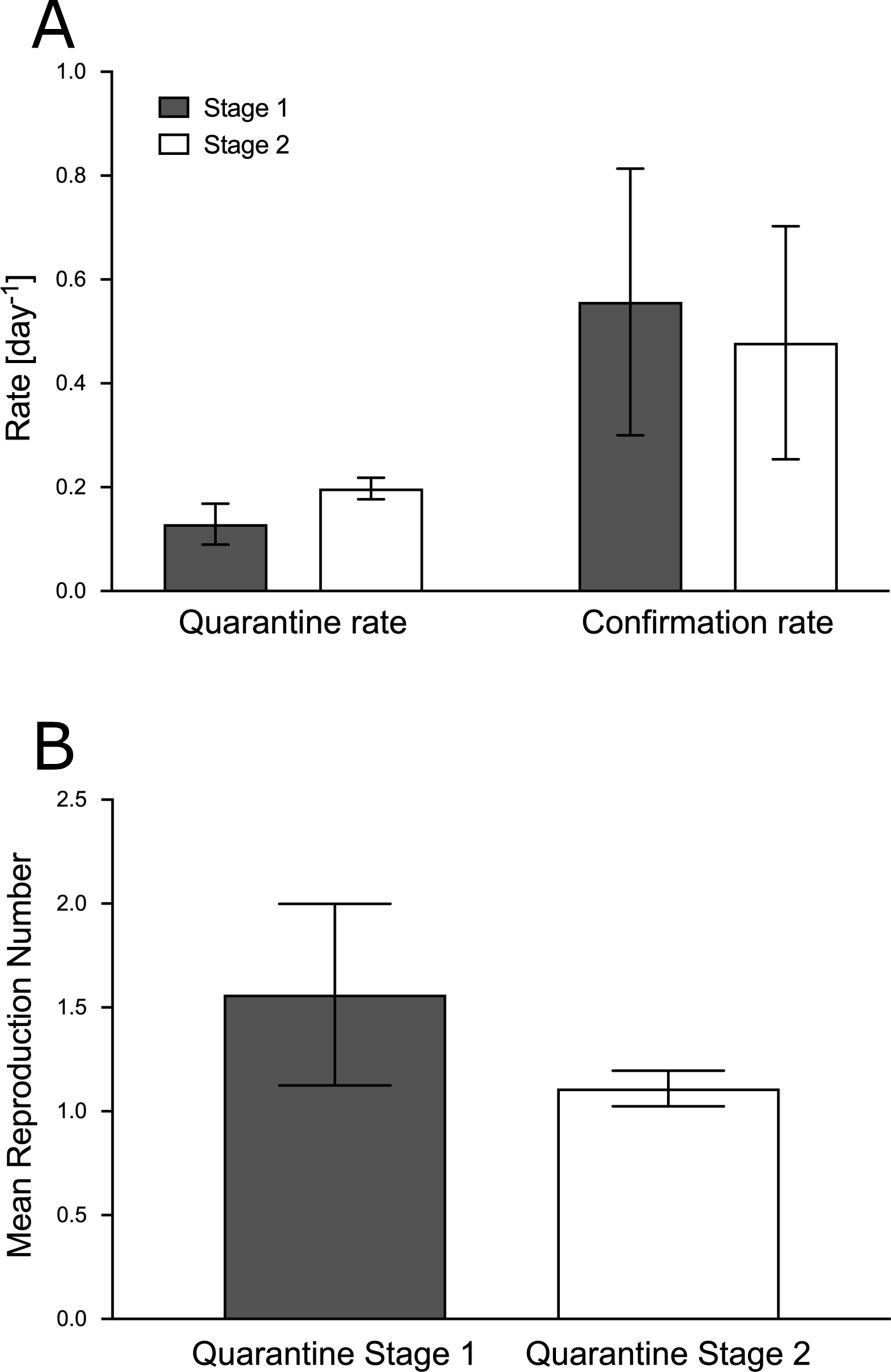
Comparison of mean goodness-of-fit of epidemiologically relevant parameters. The mean values per quarantine stage of the goodness-of-fit of the 32 communes of the province of Santiago, Chile, are compared for stages 1 and 2: quarantine rate (*γ*), confirmation rate (*β*) and reproduction number (ℜ, obtained from *γ* in Eq. (9)). Student’s *t*-test was applied to the mean values of two paired samples, in two-tailed tests, assessing *p* < 0.01 for *γ* and ℜ, and *p* = 0.23 for *β*. The values of the parameters used for calculations are outlined in Table 2.

Figure 3 shows a statistically significant increase in mean *γ* (quarantine rate), and thus, a statistically significant decrease in mean ℜ (reproduction number) from stage 1 to 2. In contrast, the mean *β* (total confirmation rate) showed no statistically significant change between stages.

**Figure 4:**
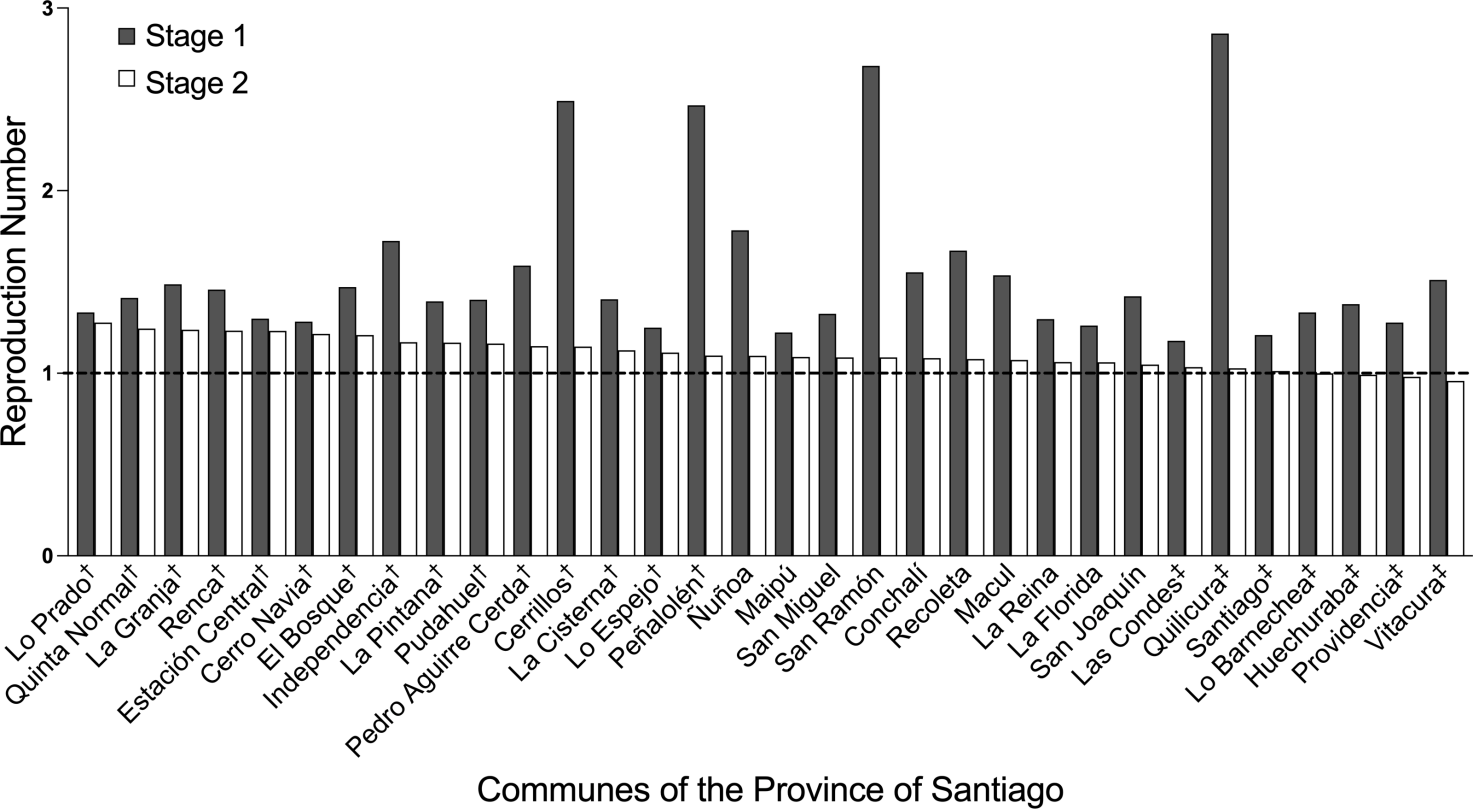
Comparison of reproduction numbers. **ℜ** values for each of the 32 communes in stages 1 and 2 are showed. From Table 2, and in qualitative terms (Seremi de Desarrollo Social y Familia Metropolitana, 2020) for the stage 2, the communes with the 15 highest ℜ values (†) had medium or high social priority (1.0958 ≤ ℜ ≤ 1.2758, 64.22 ≤ SPI ≤ 89.29). For the narrow intermediate range of ℜ (1.0324 ¡ ℜ ¡ 1.0958), corresponding to 10 communes, the social priority was of any kind. While the seven lowest ℜ values (‡) were from communes without or with low social priority (0.9571 ≤ ℜ ≤ 1.0324, 7.94 ≤ SPI ¡ 64.22).

Figure 4 shows the ℜ values for each of the 32 communes in stages 1 and 2 (Table 2). From Fig. 4 and Table 2, in the stage 2 and in qualitative terms, the communes with the 15 highest ℜ values had medium or high social priority (64.22 ≤ SPI ≤ 89.29, 1.2758 ≥ ℜ ≥ 1.0958). While the seven lowest ℜ values were from communes without or with low social priority (7.94 ≤ SPI <64.22, 0.9571 ≤ ℜ ≤1.0324). For the narrow intermediate range of ℜ (1.0324 < ℜ < 1.0958), corresponding to the 10 remaining communes, the social priority was of any kind.

**Figure 5:**
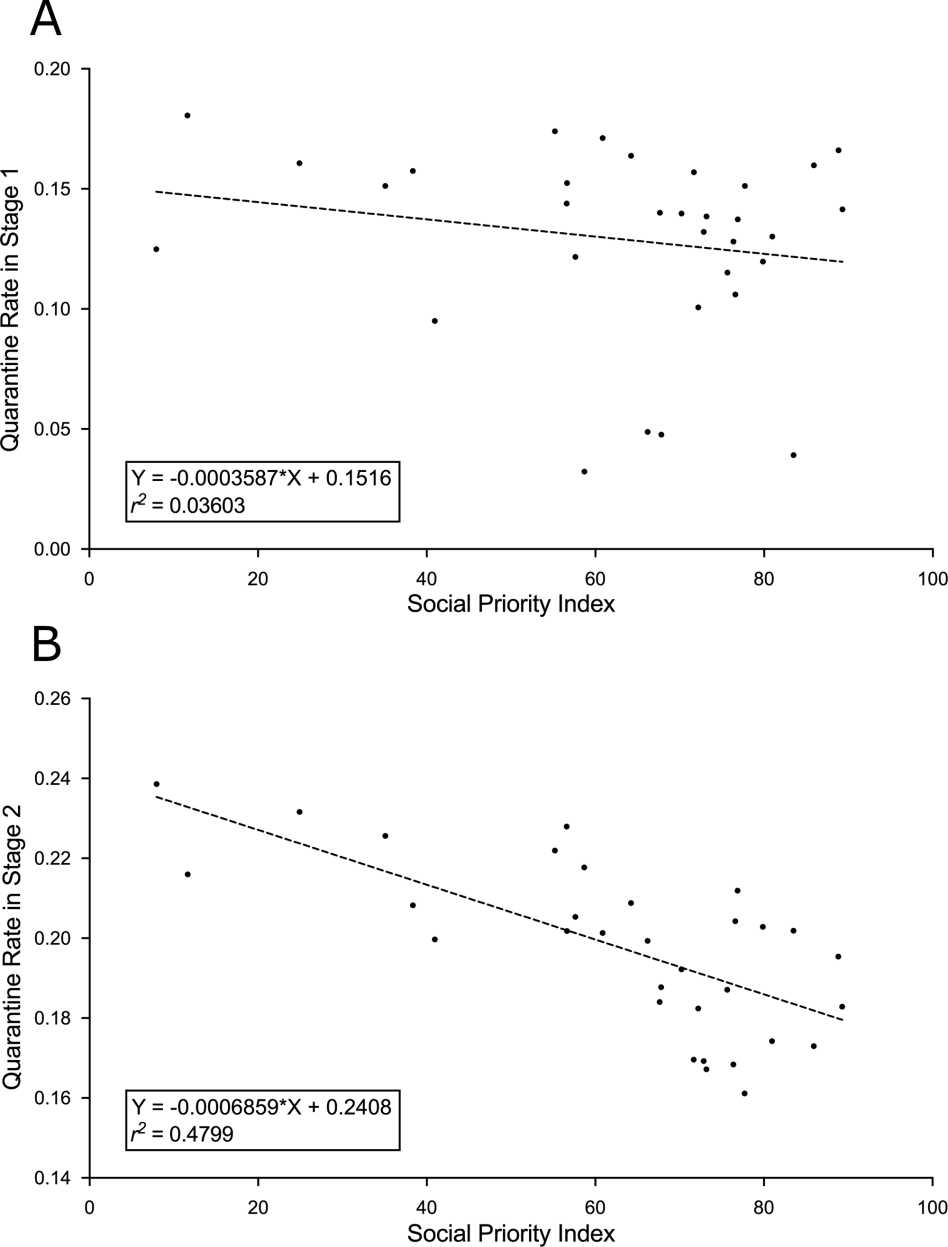
Reproduction number *versus* social priority index. The graphs indicate the regression equations and the square of the Pearson’s correlation coefficient (*r*^2^) between the quarantine rate (*γ* [day^−1^]) and the social priority index (SPI for communes in 2020) of the study communes. (A) Without quarantine imposed by the health authorities (stage 1). (B) With quarantine imposed by the health authorities (stage 2). The values of the parameters are outlined in Table 2.

For the correlation between *γ versus* SPI in stages 1 and 2, using the values outlined in Table 2, *r*^2^ was 0.0360 and 0.4799, respectively (Fig. 5).

### Assessing the robustness of results

To assess the robustness of the parameter fitting results, the following Montecarlo experiment was performed: Gaussian 100 *ϵ* % noise was added to data record of the confirmed daily incidences in a commune (*η*(*t*)), and new data replacing *η*(*t*) were defined as ${\eta }_{noisy} \left( t \right) =\eta (t)\times (1+\epsilon \mathfrak{N} \left( 0,1 \right) )$, given that $\mathfrak{N} \left( 0,1 \right) $ is a random number from the normal distribution with mean 0 and standard deviation 1. Total confirmed daily cases in the presence of noise was calculated as ${C}_{noisy} \left( t \right) ={\mathop{\sum }\nolimits }_{i={t}_{0}}^{t}{\eta }_{noisy} \left( i \right) $, determining the *β*, *γ* and ℜ adjustment parameters for each commune and in the corresponding quarantine stage (1 or 2). According to different noise levels (*ϵ*), there were 30 independent repetitions, from which means and standard deviations for above mentioned parameters were calculated. Finally, by applying the Student’s t test for paired samples from the same commune in different quarantine states, it was demonstrated that the differences between the obtained values of the same parameters are significant (*p* < 0.05) regardless of the used noise levels (*ϵ* = 0, 0.01 and 0.05).

On the other hand and following the previous experiment in case of 5% noise (*ϵ* = 0.05) on the original data, to assess the significance of the effects reported in A and B in Fig. 5, we have calculated the mean ($\overline{{\gamma }_{i}}$) and the variance (${\sigma }_{i}^{2}$) of *γ* per commune (*i* = 1, 2,…,32) and quarantine stage (1 or 2). With these statistical parameters, a lineal fitting for $\overline{{\gamma }_{i}}$ *versus* SPI_*i*_ was calculated by weighted least-squares method (Matlab curve fitting app was used, where X_data_, Y_data_ and weights were SPI_*i*_, $\overline{{\gamma }_{i}}$ and reciprocals of the variance values for each of the 32 communes, respectively). This adjustment was in cases of quarantine stages 1 and 2. In both stages, the obtained *r*^2^ (*r*^2^ = 0.228 and *r*^2^ = 0.511, for stages 1 and 2, respectively) were higher than the corresponding values obtained with the original data, ratifying that differences between the adjustment in A and B in Fig. 5 qualitatively does not vary for small levels of Gaussian noise (5%) applied on daily incidence data.

## Discussion

Along the period of the COVID-19 pandemic, Chile has been singled out as one of the countries with relatively successful control based on its spread, virulence, and lethality indicators associated with SARS-CoV-2 and its genomic variants (Canals et al., 2020; Castillo et al., 2020; Mallah et al., 2021; Sauré et al., 2022). An important finding from the Chilean experience has been that, when localized lockdowns are applied, coordination across interconnected geographic areas is necessary to reverse the growth of disease transmission produced by indirect effects from neighboring areas (Li, Undurraga & Zubizarreta, 2022). In this regard, the lack of people flux between communes in our model could affect some results. The model SUQCR assumes that contagion occurs within the same commune, which is not necessarily this case. Within the greater Santiago area, it is frequent that individuals commute for work or study reasons. Importantly, this can occur from vulnerable areas to wealthy and industrial ones. On a larger geographic scale, it has been seen that interventions in one region can affect the dynamics of the disease in another region, depending on the intensity of the interaction between the two (Freire-Flores et al., 2021). We believe that these effects due to the flow of people may affect the estimation of fitting parameters, but that the effect would be greater in stage 1 than in stage 2, since a commune in official quarantine has less interaction with other communes.

Because the effects of vaccination were not included in the mathematical model used in this study, we selected data from a period before COVID-19 vaccination, in an early stage of the pandemic in Chile. The study period was the same for all communes, with the only possible differences being the starting dates of the quarantine stages imposed by the health authorities (*i.e.,* stage 2). In turn, all the communes chosen for this study are from the same region, and therefore, are highly homogeneous, with respect to climatic conditions, with their main differences in socio-demographic parameters, such as quality of life and access to healthcare. Since there was no vaccination in the period studied and the observation period was brief, no substantial changes in the parameters are expected due to waning immunity, seasonality and variants.

In this study, we extended a known mathematical model of SARS-CoV-2 spread (SUQC model, Eqs. (1), (2), (3) and (4)) (Zhao & Chen, 2020), proposing a model that considers recovered patients, and therefore non-infectious (SUQCR model, Eqs. (5), (6), (7) and (8)). We applied this model to determine key parameters for analysis, planning, and evaluation of social distancing and quarantine measures in the current COVID-19 pandemic. The modeling data used was the time series of the total number of confirmed cases of infection with SARS-CoV-2 from each commune of the province of Santiago, Chile. For this purpose, in each commune, two consecutive social distancing and quarantine stages were considered: the first was spontaneous, not officially mandated, and the second was the quarantine imposed by the health authorities, resulting in the typical goodness-of-fit and epidemiologically relevant parameters in each stage and commune. Of all the fitting parameters calculated in this study, we were especially interested in *γ*, owing to its utility in calculating ℜ, which also depended on *α* and *D*, both constant terms in this study. Statistically significant differences were found in the mean of *γ* (increasing) and the mean of ℜ (consequently decreasing, by Eq. (9)) between the two quarantine stages. Instead, no statistically significant differences were found in the mean of *β* between the same two quarantine stages, in line with the maintenance of protocols and with the detection techniques for infected patients.

In SUQCR model, despite being in quarantine, the contacts within the household may be unavoidable, and given that quarantined individuals can also decide not to follow the instructions, generating offspring infections (Ashcroft, Lehtinen & Bonhoeffer, 2022; Kerr et al., 2021; Kretzschmar, Rozhnova & Van Boven, 2021), the ideal binary condition in which infected individuals are necessarily U or Q must be corrected by continuous values of gamma, *i.e.*: although gamma is the quarantine rate, its values within the interval [0, 1] can account for the quality of the quarantine, and the more incomplete, partial, defective, or ineffective a quarantine regime is, the lower the gamma value. In this way, communes with higher SPI (*i.e.,* lower gamma in stage 2) should have quarantines of lower quality and its consequences. According to this, it has been reported that the poor quality of housing favors the spread of COVID-19 in the province of Santiago (Vergara-Perucich, Correa-Parra & Aguirre-Nuñez, 2020). Besides, other factors that decrease the quality of quarantines, such as greater mobility associated with lower-income communes, or socioeconomic inequalities between individuals, can also increase the incidence of the disease (Bennett, 2021; Mena et al., 2021).

The quarantine measures imposed by the health authorities increased *γ* in all the communes studied. The increases in *γ* and the corresponding decreases in ℜ occurred in the first days of the quarantines in the stage 2, with characteristic values for the entire period. We believe that the study communes were already applying widely publicized self-care health recommendations during stage 1, such as avoiding crowds and closed environments, wearing a mask, and hand washing. The initial news about the beginning of the COVID-19 pandemic emerged from China in January 2020, and the first cases of COVID-19 in Chile were detected in early March 2020, when some more extensive health measures were implemented in airports and in some communes. In turn, assuming all study communes made similar efforts to comply with the imposed quarantines (stages 2) should facilitate the interpretation of differences in their results during this stage. Accordingly, the differences between the values of ℜ in stage 2 likely resulted from structural, environmental, and social deficiencies in each commune.

Furthering the above, each commune is characterized by a set of environmental, cultural, economic, demographic, and population (mental and physical) health variables, which could influence the potential to comply with the social distancing or quarantine measures imposed by the health authorities in stage 2 and by the effectiveness of its compliance. As such, the relationship between SPI of a commune, an indicator of its socioeconomic deficiencies, and ℜ is interesting. Our results showed that under imposed quarantine, *γ* tends to increase as SPI decreases in the communes. In fact, the negative correlation between the *γ* and the SPI was stronger in stage 2 than in stage 1 (with *r*^2^ values of 0.4799 and 0.0360, respectively). In stage 2, communes without or with low social priority tended to avoid exponential growth more efficiently (ℜ ≈1 or ℜ < 1) than the other communes. These findings confirm that worse socio economic conditions make it more difficult to comply with or decrease the effectiveness of the pandemic control measures, such as social distancing and quarantines (Villalobos Dintrans et al., 2021). The correlation between *γ* and SPI was much weaker in stage 1, possibly due to the lack of imposed quarantine and self-care behavior of the population of the communes, which was rather spontaneous.

Our results align with those by Mena et al. (2021), who studied human mobility (as well as health system characteristics) across communes in the Greater Santiago area and established a correlation between socioeconomic status and COVID-19-related mortality. They used measures of human mobility inferred from anonymized mobile phone data as a proxy for social distancing, whereas our study estimated effects of such social distancing. Although our model does not consider mobility data, using the model presented here to study the effect of nonpharmacological interventions could provide insights on different socioeconomic determinants of the outcome of a pandemic. Furthermore, one additional factor of inequity in Chile is its marked centralization, where access to healthcare and other services is highly concentrated in the capital city in detriment to all the rest of the country. In this sense, mathematical simplicity is an advantage for a model to be used in comparison of different regions or contexts, and this deterministic SUQCR model has been shown to predict the dynamics of COVID-19 by means of a small number of parameters of epidemiological importance. However, since its applications in the analysis and control of epidemics can influence public policies, this study must be completed with sensitivity analysis, considering different sources of error, to provide results with confidence intervals.

## Conclusions

More precise or similar relationships could be calculated using a more extensive and varied database of epidemiological parameters. Accordingly, different countries should have similar epidemiological models and share their databases on the relevant characteristics in each commune or region, this to be able to increase the necessary social distancing and compliance with the quarantines. These measures are the main non-invasive interventions that have proved useful in reducing the spread of COVID-19. In fact, the effect of the imposed lockdown measures in the communes appeared noticeably early. Conversely, given the negative impacts of these extreme measures on quality of life and economic activity, these interventions should be planned and evaluated carefully. For these reasons, the model applied here may significantly help to identify determining factors of the success of these external intervention measures, facilitating their control, monitoring, and evaluation.

##  Supplemental Information

10.7717/peerj.14892/supp-1 Supplemental Information 1 Programs (codes, input data and instructions)

10.7717/peerj.14892/supp-2 Supplemental Information 2 Raw data of stages 1 and 2

## Additional Information and Declarations

### Data Availability

The following information was supplied regarding data availability: The data and code are available in the Supplemental Files.
